# LIVES WORSE THAN DEATH: ADULT CHILDREN ARE POOR AT ESTIMATING PARENTS’ QUALITY-OF-LIFE VALUATIONS

**DOI:** 10.1093/geroni/igz038.2368

**Published:** 2019-11-08

**Authors:** Matthew Pichiello, Meghan McDarby, Elissa K Kozlov, Brian Carpenter

**Affiliations:** 1 Washington University in St. Louis, St. Louis, Missouri, United States; 2 Rutgers University, New Brunswick, New Jersey, United States

## Abstract

Adult children often help older parents make medical decisions when their health is compromised. To do so in a way that respects parent values requires children to know how their parent views health states and consequent quality of life. The current study compared older parent and adult child valuations of quality of life in different health contexts. Families consisted of older parents (n = 37) and their adult children (n = 66). Parents rated perceived quality of life in 14 compromised health states on a scale from 1 (difficult but acceptable) to 5 (not worth living). Children estimated how their parent responded to each health state, yielding an index of their knowledge of parent perceptions. Overall, parents described all compromised health states as less acceptable than adult children thought they would, t(99) = 2.19, p < .05. Notably, parents believed situations that caused financial or emotional burden to their family were much less acceptable than their children estimated. Children were more knowledgeable about parent valuations for more extreme circumstances, such as living with a feeding tube. Within families, children demonstrated only slight knowledge about parent quality of life valuations (kappa = .081). Across the entire sample of families, there was a broad range of knowledge (kappas = -.181 – .351), but at best, knowledge was still only fair. Results from this study suggest that adult children may underestimate the impact of compromised health states on parent estimations of quality of life, which could affect collaborations on healthcare decisions.

